# Identification of inflammatory phenotypes of asthma by blood analysis and clinical parameters

**DOI:** 10.1186/2045-7022-3-S1-O9

**Published:** 2013-05-03

**Authors:** Bart Hilvering, Rene Schweizer, Jan-Willem Lammers, Leo Koenderman

**Affiliations:** 1 UMC Utrecht, the Netherlands; 2 UMC Utrecht, Respiratory Medicine, the Netherlands

## Background

Identification of an inflammatory asthma phenotype currently requires sputum induction. This technique is invasive, has a high variability, is time-consuming and a burden for patients. Therefore, there is a strong need for a routine blood test to establish the inflammatory phenotype of asthma. We designed a clinical cohort study (AIR-study, NCT01611012) in 115 asthma patients visiting the outpatient clinic to compare the results of such a blood test to sputum analysis. This abstracts focuses at the preliminary results in 21 patients.

## Objective

To assess whether expression of active FcgammaRII (CD32) and MAC-1( CD11b) on neutrophils and eosinophils in peripheral blood enables the diagnosis of an inflammatory phenotype of asthma.

## Materials and methods

21 asthma patients were recruited at the outpatient clinic of the University Medical Centre Utrecht. Clinical parameters were gathered and FENO (fractional exhaled nitric oxide), sputum induction and blood tests were performed. Eosinophils and neutrophils in whole blood were stained with a FITC labeled antibody against active FcgammaRII receptor (clone A17) and a PE-labeled antibody against CD11b in the absence and presence of the activator fMLP (1 µM). Subsequently, fluorescence intensity was measured by flowcytometry.

## Preliminary results

Expression of Mac-1 (CD11b) and activeFcgammaRII (CD32) on eosinophils at basal level and after stimulation (fMLP) showed refractory cells (low responsiveness for fMLP) in case of granulocytic asthma (neutrophilic, eosinophilic and mixed phenotype). FENO values were overall lower in paucigranulocytic asthma compared to granulocytic asthma (p = 0.009, independent t-test).

## Conclusion

Refractory blood eosinophils determined by a simple blood test were found in those patients that were more likely to suffer from granulocytic asthma (diagnosis by sputum analysis). Therefore, paucigranulocytic asthma can be distinguished from other phenotypes purely based on blood analysis and FENO measurement. A low expression of activation epitopes on eosinophils in peripheral blood might indicate that **‘**activation prone**’** eosinophils migrated to the lung.

